# Association of computed tomography‐derived muscle mass and quality with delayed acquisition independent walking after cardiovascular surgery

**DOI:** 10.1002/jcsm.13521

**Published:** 2024-06-19

**Authors:** Kazuya Shimizu, Ryota Matsuzawa, Shinya Nakamura, Keita Murakawa, Hideo Kawakami, Masaki Tabuchi, Motoaki Ohnaka, Masamichi Matsumori, Akira Tamaki

**Affiliations:** ^1^ Department of Rehabilitation Sumitomo Hospital Osaka Japan; ^2^ Course of Rehabilitation Science Graduate School of Rehabilitation Science, Hyogo Medical University Kobe Japan; ^3^ Department of Physical Therapy, School of Rehabilitation Hyogo Medical University Kobe Japan; ^4^ Department of Cardiovascular Surgery Sumitomo Hospital Osaka Japan

**Keywords:** Cardiac rehabilitation, Cardiovascular surgical procedure, Early ambulation sarcopenia, Frailty, Preoperative exercise

## Abstract

**Background:**

In the context of cardiovascular surgery, the foremost concern lies in delayed functional recovery, as typified by the acquisition of independent walking after surgery, among older patients with decline in skeletal muscle mass and quality. Computed tomography (CT), which is typically employed for the preoperative assessment of pathological conditions in patients undergoing cardiovascular surgery, is also suitable for screening for potential decline in skeletal muscle mass and quality. The aim of this study was to examine the predictive capabilities of CT‐derived parameters such as muscle mass and muscle quality for the delayed acquisition of independent walking in the postoperative period.

**Methods:**

This retrospective study enrolled consecutive Japanese patients who underwent elective cardiovascular surgery between May 2020 and January 2023. In total, 139 patients were included in the analyses. Based on the preoperative CT image, the psoas muscle volume index (PMVI) and psoas muscle attenuation (PMA) were calculated. Information on patient characteristics, including preoperative physical fitness such as handgrip strength/body mass index (GS/BMI), short physical performance battery (SPPB), and 6‐min walking distance (6MWD), were obtained from the medical records. We defined delayed acquisition of independent walking after surgery as the inability to walk 100 m within 4 days after surgery.

**Results:**

The median age of the patients was 72 (interquartile: 64–78) years, and 74.8% (104/139) were men; 47.5% corresponded to the delayed group. The areas under the curves of SPPB, GS/BMI, 6MWD, PMVI, and PMA against delayed acquisition of independent walking after surgery were 0.68 [95% confidence interval (CI): 0.59 to 0.77], 0.72 (95% CI: 0.63 to 0.80), 0.73 (95% CI: 0.65 to 0.82), 0.69 (95% CI: 0.60 to 0.78), and 0.78 (95% CI: 0.70 to 0.85), respectively. In the multivariate logistic regression analysis, low PMA was significantly associated with delayed acquisition of independent walking even after adjustment for patient characteristics including physical fitness [model 1: SPPB (OR, 1.14; 95% CI: 1.03–1.25), model 2: GS/BMI (OR, 1.13; 95% CI: 1.03–1.25), and model 3: 6MWD (OR, 1.14; 95% CI: 1.03–1.25)], but PMVI was not.

**Conclusions:**

Our study revealed a strong association between PMA, a marker of CT‐derived muscle quality, and the postoperative delay in achieving independent walking in patients who underwent cardiovascular surgery. The technique to obtain information on muscle quality during the time period before surgery may be an option for timely therapeutic intervention in patients who may have delayed acquisition of independent walking after surgery.

## Introduction

Sarcopenia is a geriatric syndrome characterized by a progressive decline in skeletal muscle quantity and quality, which renders individuals vulnerable to exogenous stress and significantly increases their susceptibility to adverse health consequences.^1^, ^2^ In the context of cardiovascular surgery, the foremost concern lies in delayed functional recovery, as typified by the acquisition of independent walking after surgery, among older patients with decline in skeletal muscle mass and quality.^3^, ^4^ Such a postponement in achieving independent ambulation after surgery inevitably leads to prolonged hospitalization and an increased risk of mortality and readmission.^5^, ^6^, ^7^, ^8^ Computed tomography (CT) is considered the gold standard for evaluating skeletal muscle mass, and recently, it has been used as a tool for precise evaluation of muscle quality.^1^ This insight has sparked the concept of utilizing pre‐existing CT images not only to assess preoperative pathological conditions but also to gauge the risk of decline in skeletal muscle mass and quality.^9^ Despite recent studies on CT‐derived skeletal muscle assessment within the domain of cardiovascular surgery,^10^, ^11^, ^12^ research specifically investigating its predictive capacity for delayed acquisition of independent ambulation after these types of surgeries remains scarce. A few reports have examined the association between preoperative muscle mass and muscle function with the delayed attainment of independent ambulation following cardiovascular surgery.^3^, ^4^, ^13^, ^14^ However, there is a conspicuous lack of research that has evaluated the predictive potential of preoperative CT‐derived skeletal muscle parameters for the outcome.

Hence, the primary objective of this study was to assess the predictive capacity of muscle mass and quality, as calculated from preoperative CT images, for the delayed attainment of independent gait following cardiovascular surgery. Notably, the use of CT parameters may significantly enhance the postsurgical risk stratification process and could serve as valuable preoperative therapeutic targets in the context of cardiac rehabilitation.

## Methods

### Study design and participants

In total, 178 consecutive Japanese patients who underwent elective thoracic cardiovascular surgery, including coronary artery bypass graft, valvular disease surgery, aortic disease, or combined surgery, at our hospital between May 2020 and January 2023 were enrolled and assessed for their eligibility for inclusion in this retrospective study. Patients with transverse abdominal CT images obtained before elective cardiovascular surgery and those who underwent in‐hospital cardiac rehabilitation were included. The exclusion criteria were as follows: (i) diagnosis of dementia; (ii) inability to walk independently before surgery; (iii) inability to transition to a cardiac rehabilitation programme such as walking training or aerobic exercise due to severe postoperative complications such as stroke, respiratory failure, sepsis, severe heart failure, or infection; (iv) in‐hospital death; or (v) missing data. The study was announced using a poster informing patients that their health‐related data from the centre would be anonymized and provided to the study. Although the study participants did not provide written or verbal informed consent, they were allowed to refuse or withdraw their participation at any time. This opt‐out procedure conformed to the Japanese Ethical Guidelines for Epidemiological Research for observational studies using existing data. The study protocol, including the consent procedure, was approved by the Ethics Committee of our hospital (approval number: 2023‐8) and conducted in accordance with the principles of the Declaration of Helsinki.

### Patient characteristics

Information on demographic factors (age and sex), physical constitution [body mass index (BMI)], type of surgery, co‐morbid conditions (hypertension, dyslipidaemia, and diabetes mellitus), smoking habits, laboratory data [C‐reactive protein (CRP), white blood cell (WBC), serum albumin level, haemoglobin level, serum creatinine level, estimated glomerular filtration rate (eGFR), and brain natriuretic peptide (BNP)], and echocardiographic parameters [left ventricular ejection fraction (LVEF), early mitral inflow velocity to mitral annular early diastolic velocity (E/e′), left ventricular internal dimension in diastole (LVDd), and left ventricular internal dimension in systole (LVDs)] was collected. Geriatric nutritional risk index (GNRI) was calculated based on serum albumin level and BMI as an index of nutritional condition.^15^ Physical fitness, including handgrip strength (GS), short physical performance battery (SPPB), and 6‐min walking distance (6MWD), was measured on the day before surgery and obtained from medical records.

The GS was measured twice on each side using a Smedley digital dynamometer (TKK 5101 Grip‐D; Takei, Tokyo, Japan) according to a standard protocol.^16^, ^17^ The Smedley dynamometer has exhibited a strong correlation (*r* = 0.94) with the JAMAR Hydraulic Hand dynamometer.^18^ Furthermore, it underwent calibration prior to the assessments. Maximal isometric voluntary contractions of the hands for 3 s each were assessed two‐trial with the patients in the standing position and full elbow extension. The highest strength values on the right and left sides were averaged, transformed into kilograms, and adjusted for BMI.^19^ The SPPB, which consists of three items: gait speed, standing balance, and five‐time chair stand time, was measured using established methods.^1^, ^20^ The SPPB scores ranged from 0 to 12, that is, 0–4 points for each component (0 = worst, 12 = best). The 6MWD was measured according to the guidelines established by the American Thoracic Society,^21^ and the participants were requested to walk as far as possible for 6 min. The patients used their usual walking aids during the test. These assessments of physical fitness were conducted by trained physical therapists, who followed manuals created based on guidelines,^1^, ^16^, ^17^, ^21^ containing instructions such as orientation and verbal cues, striving to ensure consistency in methods among different assessors.

After surgery, information on the surgical procedures (operating time, cardiopulmonary bypass duration, aortic cross clamping time, and volume of bleeding), postoperative complications (delirium, atrial fibrillation, stroke, respiratory failure, sepsis, severe heart failure, and infection), durations requiring mechanical ventilation and spent in the intensive care unit, and number of days from surgery to the start of out‐of‐bed care were obtained from the surgical or medical records. Delirium assessments were conducted every 8 h during the ICU stay by trained bedside nurses after surgery using the Confusion Assessment Method for the Intensive Care Unit (CAM‐ICU).^22^ The assessment of delirium was also checked retrospectively by research staff using the patients' medical records.

### Computed tomography image analysis

**Figure 1:**
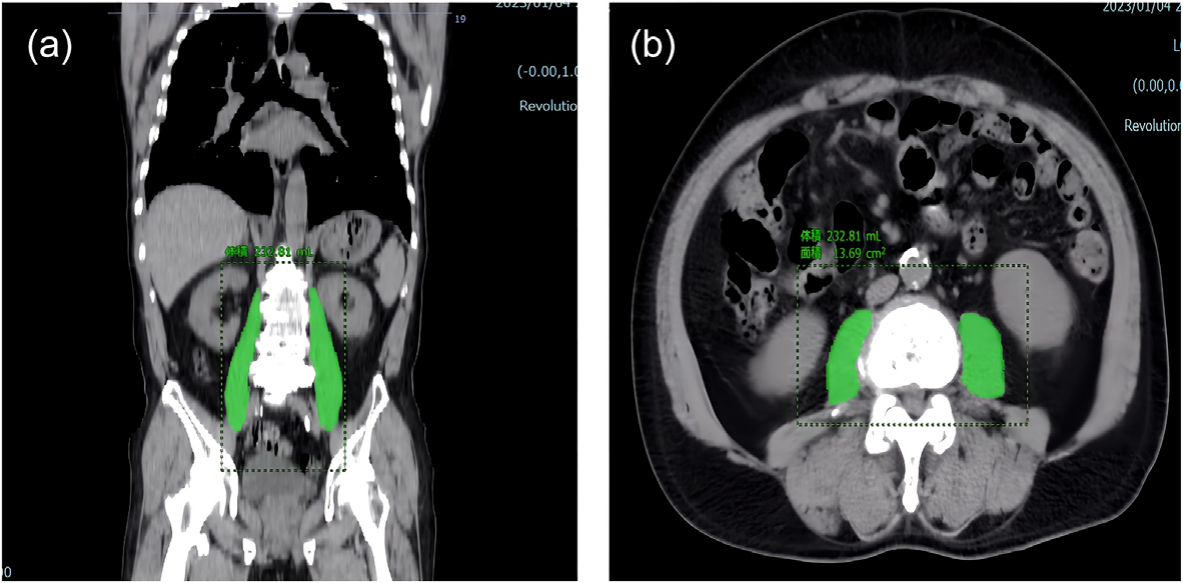
Assessment of the psoas muscle on CT images. Psoas muscle volume in the coronal (A) and axial (B) planes.

Skeletal muscle volume and attenuation were assessed using the most recent preoperative CT scan of each patient. The psoas muscle volume (PMV) was calculated automatically using a deep learning‐technology‐based image processing programme (Synapse SAI Viewer, Fujifilm, Tokyo, Japan). The tube potential was set between 100 and 120 kV with a 512 × 512 matrix, and the electrical current (mA) was set automatically for each slice. The slice thickness was set to 5 mm, and contrast enhancement was performed in the non‐contrast scan or unenhanced phase. Patients without non‐contrast scans were excluded because contrast significantly increases the CT muscle density.^23^ Figure 1 shows the PMV in the axial and coronal images. PMV on the left and right sides were summed and divided by the square of the height of the patient and expressed as the psoas skeletal muscle volume index (PMVI). Psoas muscle attenuation (PMA) was quantified as the mean muscle attenuation (MA) of the psoas cross‐sectional area (CSA) across the lower border of the L3 vertebra.^24^ Measurements were performed by two independent investigators. Before conducting this study, the researchers received instructions from a professional radiologist on how to identify the lower border of the L3 vertebra. Inter‐ and intraobserver agreements were assessed using intraclass correlation coefficients by analysing 10 randomly chosen patients by two independent investigators. The inter‐ and intraobserver intraclass correlation coefficients were excellent (0.922, 0.930).^24^, ^25^ To avoid measurement bias, the measurements were conducted in a blinded fashion, wherein the participants could not be identified. Furthermore, these procedures were performed at well‐timed intervals after the patient was discharged.

### Inpatient cardiac rehabilitation

The postoperative rehabilitation programme at our hospital was based on the Guidelines of the Japanese Circulation Society,^26^ which aim to achieve walking independence within 4 days after surgery. The criteria for initiating out‐of‐bed care and stepping up the programme were in line with the guidelines and consistent among the therapists. Briefly, patients practiced one or two sets of stepping at the bedside after sitting at the edge of the bed for 10 min on the first and second postoperative days, and if cleared, proceeded to walking exercises within the intensive care unit. According to the criteria of the guidelines, patients started to get out of bed if the following conditions are ruled out: (1) low output syndrome (LOS), (2) insertion of a Swan‐Ganz catheter, (3) resting heart rate ≥120 b.p.m., (4) unstable blood pressure (blood pressure drops only with positional changes), (5) unstable haemodynamics, and (6) unstable pulse rate. In addition, after the start of the weaning process, the exercise protocol for the patient proceeded step by step while checking the step‐up criteria as follows: (1) no chest pain, severe shortness of breath, strong fatigue (Borg scale >13), dizziness, lightheadedness, or leg pain, (2) no cyanosis, pallor or cold sweat, (3) no tachypnoea (≥30 breaths/min), (4) no increase in arrhythmia or rhythm change to AF with exercise, (5) no ischaemic ECG change with exercise, (6) no excessive blood pressure changes with exercise, (7) no increase in HR by more than 30 beats/min with exercise, and (8) no decrease in arterial oxygen saturation to <90% with exercise. The walking distance extension programme started with an indoor walk, extended to a 50 m walk, and then to a 100 m walk, although the programme was designed and modified appropriately according to the physical function and general condition of each individual.

### Outcome

The primary outcome was whether the patients could walk independently within 4 days after surgery. The 4‐day walk test is recommended by the Japanese guidelines based on evidence from a multicentre survey in Japan that reported that it took 3.8 days for patients to regain the ability to walk independently after successful elective cardiac surgery.^26^ The definition of independent walking in this study was 100 m of walking without assistance or supervision from another person, although the patient could use an assistive device (e.g. a cane) if necessary. Walking ability was assessed by physical therapists with no information on the PMV and PMA calculated using CT image analysis before surgery.

### Statistical analysis

Data are expressed as means ± standard deviation (SDs) or median with interquartile range for continuous variables, and as numbers and percentages for categorical variables. The patients were classified into two groups according to the progress of cardiac rehabilitation: the no delay group included patients who achieved 100 m of independent walking within 4 days after surgery, and the delay group included patients who were unable to achieve this. The patient characteristics were compared between the groups using an unpaired *t*‐test or the Mann–Whitney *U* test for continuous variables and the χ^2^ test for categorical variables. To calculate the areas under the curves (AUCs) and cutoff points of the discriminant parameters for predicting delayed acquisition of independent walking, we performed receiver operating characteristic (ROC) curve analyses. We used the Youden index^27^ to determine the cutoff points of each parameter for predicting the acquisition of independent walking. The Youden index is used as a measure of the overall combined specificity and sensitivity of a predicting factor and is defined as the maximum vertical distance between the ROC curve and the diagonal of the chance line, and is calculated as maximum (sensitivity + specificity − 1). To evaluate the association of PMVI and PMA with the acquisition of independent walking, after adjusting for potential confounders, including age, BMI, type of operation, GNRI, eGFR level, BNP level, delirium, duration of mechanical ventilator use, SPPB, GS/BMI, and 6MWD, we conducted univariate and multivariate logistic regression analyses. Odds ratios (ORs) per 1‐point change and 95% confidence intervals (CIs) were calculated. Statistical analyses were performed using SPSS software version 27 (IBM, New York, USA). Statistical significance was set at *P* < 0.05.

## Results

### Patient characteristics

**Figure 2:**
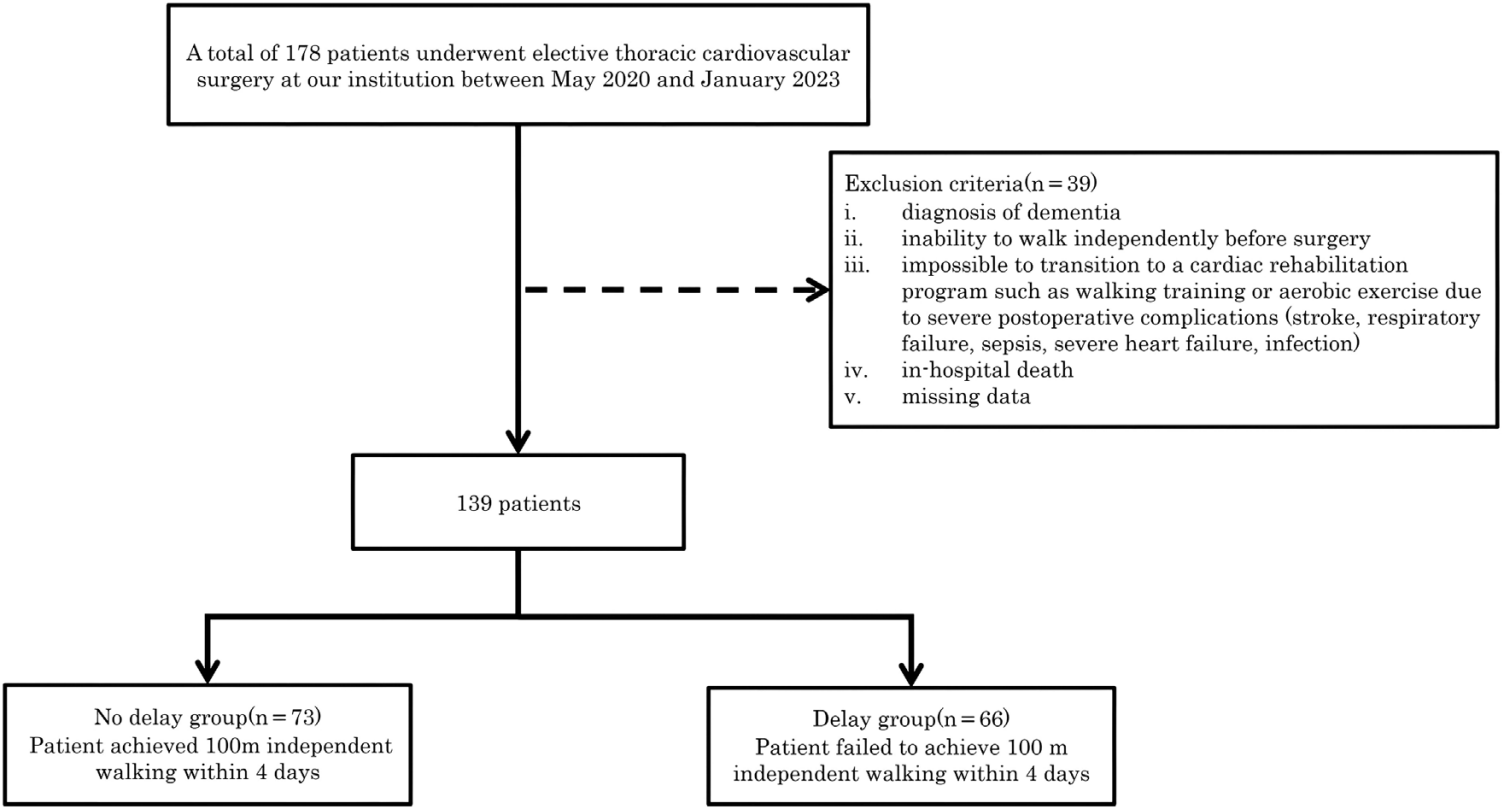
Study flow chart.

We excluded 24 (61.5%) patients who had missing characteristic data, 6 (15.4%) patients who were unable to walk independently before surgery, 4 (10.3%) patients who were unable to transition to a cardiac rehabilitation programme such as walking training or aerobic exercise due to severe postoperative complications, and 5 (12.8%) patients who died in the hospital; therefore, 139 patients were included in the final analyses (Figure 2). Patient characteristics are listed in Table 1. A total of 73 (52.5%) patients achieved 100 m of independent walking without delay. All physical fitness variables in the delayed group were significantly lower than those in the no delay group. The crude or normalized height‐squared PMVI and PMA were significantly larger in the no delay group than in the delay group (*P* < 0.001). Information on the intraoperative and postoperative clinical characteristics and progress of early postoperative cardiac rehabilitation are listed in Table 2. There were no significant differences in operative records between the two groups. In the postoperative clinical data, the delay group had higher delirium (*P* < 0.001), longer duration requiring mechanical ventilator (*P* < 0.001), and length of ICU stay (*P* < 0.001) than the no delay group.

**Table 1:** Patient characteristics

	Total (*n* = 139)	No delay group (*n* = 73)	Delay group (*n* = 66)	*P*‐value
Age (year)	72.0 [64.0, 78.0]	67.0 [57.5, 73.5]	75.5 [70.0, 80.0]	<0.001
Male (%)	104 (74.8%)	62 (84.9)	42 (63.6)	0.006
Body mass index (kg/m^2^)	23.8 [21.5, 26.7]	24.5 [22.4, 26.8]	23.2(20.9, 26.1]	0.074
Type of operation (%)				0.281
CABG	20 (14.4)	14 (19.2)	6(9.1)	
Valve	41 (29.5)	24 (32.9)	17 (25.8)	
Aortic	41 (29.5)	18 (24.7)	23 (34.8)	
Multiple	37 (26.6)	17 (23.3)	20 (30.3)	
Co‐morbid conditions				
Hypertension (%)	90 (64.7)	45 (61.6)	45 (68.2)	0.420
Dyslipidaemia (%)	67 (48.2)	37 (50.7)	30 (45.5)	0.538
Diabetes mellitus (%)	42 (30.2)	21 (28.8)	21 (31.8)	0.696
Smoking (%)				0.494
Never	83 (59.7)	42 (57.5)	41 (62.1)	
Past	48 (34.5)	26 (35.6)	22 (33.3)	
Active	8 (5.7)	5 (6.8)	3 (4.5)	
Echocardiographic parameters				
LVEF (%)	59.5 ± 12.2	58.5 ± 13.1	61.1 ± 10.8	0.409
E/e′	11.3 [8.9, 14.8]	11.3 [8.7, 14.8]	11.3 [8.9, 14.7]	0.921
LVDd (mm)	50.0 ± 8.1	52.3 ± 7.9	47.5 ± 7.6	<0.001
LVDs (mm)	31.5 [20.0, 38.0]	34.0 [29.2, 41.7]	31.0 [27.0, 34.2]	0.061
Laboratory parameters				
CRP (mg/dL)	0.12 [0.05, 0.44]	0.08 [0.04, 0.27]	0.22 [0.07, 0.81]	0.075
WBC (cells/μL)	5800 [4700, 7000]	5900 [5050, 7100]	5800 [4375, 6750]	0.728
Haemoglobin (g/dL)	12.8 ± 1.9	13.4 ± 1.8	12.0 ± 1.8	<0.001
eGFR (mL/min/1.73 m^2^)	57.0 [38.0, 68.2]	61.6 [44.4, 71.2]	46.0 [11.0, 64.0]	0.074
Creatinine (mg/dL)	0.9 [0.7, 1.4]	0.9 [0.7, 1.3]	1.0 [0.7, 3.6]	0.555
BNP (pg/mL)	52.8 [26.3, 137.7]	38.1 [20.0, 96.9]	75.5 [37.5, 230.1]	0.039
GNRI	105.3 ± 11.9	108.1 ± 10.1	102.2 ± 13.0	0.003
PMVI (cm^3^/kg/m^2^)	98.2 ± 29.1	107.5 ± 28.2	87.9 ± 26.8	<0.001
PMA (HU)	45.7 [39.7, 49.5]	48.1 [44.9, 51.5]	41.4 [35.5, 47.3]	<0.001
GS/BMI (kg/kg/m^2^)	1.1 [0.8, 1.3]	1.2 [0.9, 1.4]	0.9 [0.7, 1.1]	<0.001
SPPB	12.0 [10.0, 12.0]	12.0 [11.0, 12.0]	11.0 [9.0, 12.0]	<0.001
6MWD (m)	392.0 ± 99.2	431.8 ± 74.4	347.9 ± 104.9	<0.001

Note: Median [25th, 75th percentile], *n* (%), or mean ± standard deviation.

6MWD, 6‐min walking distance; BMI, body mass index; BNP, brain natriuretic peptide; CABG, coronary artery bypass grafting; CRP, C‐reactive protein; E/e′, early mitral inflow velocity to mitral annular early diastolic velocity; eGFR, estimated glomerular filtration rate; GNRI, Geriatric Nutritional Risk Index; GS, grip strength; HU, Hounsfield units; LVDd, left ventricular internal dimension in diastole; LVDs, left ventricular internal dimension in systole; LVEF, left ventricular ejection fraction; PMA, psoas muscle attenuation; PMVI, psoas muscle volume index; SPPB, short physical performance battery; WBC, white blood cell.

**Table 2:** Intraoperative, postoperative clinical characteristics, and progress of early postoperative cardiac rehabilitation

	Total (*n* = 139)	No delay group (*n* = 73)	Delay group (*n* = 66)	*P*‐value
Operation time (min)	278 [233, 327]	264 [217, 319]	291 [236, 343]	0.108
CPB time (min)	138 [103, 166]	129 [94, 164]	144 [115, 173]	0.352
ACC time (min)	80 [53, 110]	74 [53, 106]	89 [51, 116]	0.440
Volume of bleeding (mL)	507 [253, 827]	478 [217, 843]	517 [262, 822]	0.935
Duration requiring mechanical ventilator (min)	465 [132, 1110]	182 [110, 1049]	1030 [174, 1154]	0.001
POAF (%)	48 (34.5)	23 (31.5)	25 (37.9)	0.430
Delirium (%)	22 (15.8)	2 (2.7)	20 (30.3)	<0.001
Sitting on edge of bed (days)	1.0 [1.0, 1.0]	1.0 [1.0, 1.0]	1.0 [1.0, 1.0]	0.004
Standing at bedside (days)	1.0 [1.0, 1.0]	1.0 [1.0, 1.0]	1.0 [1.0, 2.0]	<0.001
Walking around bed (days)	2.0 [1.0, 3.0]	2.0 [1.0, 2.0]	4.0 [2.0, 4.7]	<0.001
100 m of independent walking (days)	4.0 [3.0, 6.0]	4.0 [3.0, 4.0]	6.5 [5.0, 11.2]	<0.001
Length of ICU (days)	2.0 [2.0, 4.0]	2.0 [2.0, 3.0]	3.0 [2.0, 4.0]	<0.001
Length of hospital stay (days)	19.0 [16.0, 24.0]	18.0 [15.0, 20.5]	22.0 [18.7, 29.0]	<0.001

Median [25th, 75th percentile], *n* (%), or mean ± standard deviation.

ACC, Aortic cross clamping; CPB, cardiopulmonary bypass duration; POAF, postoperative atrial fibrillation.

### Area under the curve and cut‐off points of the predicted parameters for delayed acquisition of independent walking

**Figure 3:**
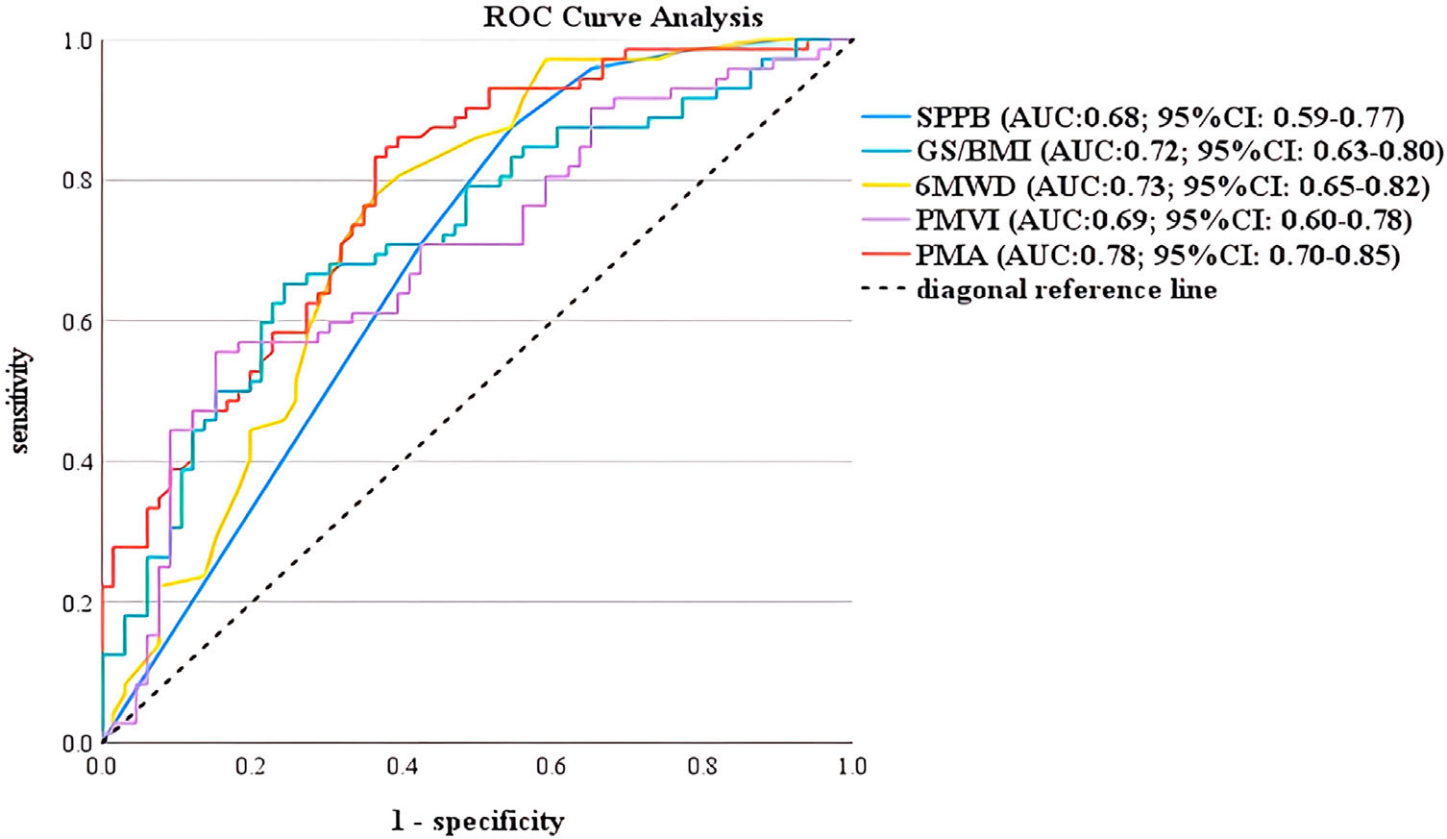
ROC curves of the performance of the predicted parameters for delayed acquisition of independent walking. 6MWD, 6‐min walking distance; AUC, area under the curve; BMI, body mass index; CI, confidence interval; GS, grip strength; PMA, psoas muscle attenuation; PMV, psoas muscle volume index; ROC, receiver operating characteristic; SPPB, short physical performance battery.

The ROC curves for the performance of the predicted parameters for the delayed acquisition of independent walking are shown in Figure 3. The AUCs of SPPB, GS/BMI, 6MWD, PMVI, and PMA against delayed acquisition of independent walking after surgery were 0.68 (95% CI: 0.59–0.77), 0.72 (95% CI: 0.63–0.80), 0.73 (95% CI: 0.65–0.82), 0.69 (95% CI: 0.60–0.78), and 0.78 (95% CI: 0.70–0.85), respectively. The predictive ability of PMA over physical fitness parameters and PMVI for delayed acquisition of independent walking was superior. The cutoff point for PMA identifying those based on the Youden index was 44.2 Hounsfield Units (HU).

### Association of the computed tomography‐derived parameters and physical function for acquisition of independent walking

The results of the univariate and multivariate logistic regression analyses are presented in Table 3. Although low PMVI was significantly associated with delayed acquisition of independent walking in the crude model (OR, 1.02; 95% CI: 1.01–1.04), after adjustment for age, type of operation, GNRI, eGFR, BNP, delirium, duration requiring mechanical ventilator, and physical fitness [model 1: SPPB (OR, 1.00; 95% CI: 0.98–1.02), model 2: GS/BMI (OR, 1.00; 95% CI: 0.98–1.02), and model 3: 6MWD (OR, 1.00; 95% CI: 0.99–1.01)], there was no association between the two sides. Conversely, low PMA was significantly associated with the elevated risk of delayed acquisition of independent walking even in the all adjusted models including age, type of operation, GNRI, eGFR, BNP, delirium, duration requiring mechanical ventilator, and physical fitness [model 1: SPPB (OR, 1.14; 95% CI: 1.03–1.25), model 2: GS/BMI (OR, 1.13; 95% CI: 1.03–1.25), and model 3: 6MWD (OR, 1.14; 95% CI: 1.03–1.25)].

**Table 3:** Association of the preoperative CT‐derived parameters and physical function for walking independence in the multivariate logistic regression analysis

	Crude model			Model 1			Model 2			Model 3		
	OR	95% CI	*P*‐value	OR	95% CI	*P*‐value	OR	95% CI	*P*‐value	OR	95% CI	*P*‐value
Muscle quality												
Short physical performance battery	1.88	1.39–2.54	<0.001	1.33	0.86–2.03	0.191	‐	‐	‐	‐	‐	‐
Handgrip strength/body mass index	14.2	4.18–48.5	<0.001	‐	‐	‐	2.35	0.44–12.5	0.317	‐	‐	‐
6‐min walking distance	1.01	1.00–1.01	<0.001	‐	‐	‐	‐	‐	‐	1	0.99–1.01	0.32
Psoas muscle attenuation	1.19	1.11–1.28	<0.001	1.14	1.03–1.25	0.006	1.13	1.03–1.25	0.010	1.14	1.03–1.25	0.006
Muscle mass												
Short physical performance battery	1.88	1.39–2.54	<0.001	1.46	1.00–2.12	0.045	‐	‐	‐	‐	‐	‐
Handgrip strength/body mass index	14.2	4.18–48.5	<0.001	‐	‐	‐	5.4	1.10–26.3	0.037	‐	‐	‐
6‐min walking distance	1.01	1.00–1.01	<0.001	‐	‐	‐	‐	‐	‐	1	1.00–1.01	0.052
Psoas muscle volume index	1.02	1.01–1.04	<0.001	1	0.98–1.02	0.427	1	0.98–1.02	0.867	1	0.99–1.02	0.395

Crude model: not adjusted for any variable. Multivariate model 1 includes age, type of operation, geriatric nutritional risk index, estimated glomerular filtration rate, brain natriuretic peptide, delirium use and duration requiring mechanical ventilator, short physical performance battery, and CT parameter (psoas muscle attenuation or psoas muscle volume index). Multivariate model 2 includes age, type of operation, geriatric nutritional risk index, estimated glomerular filtration rate, brain natriuretic peptide, delirium and duration requiring mechanical ventilator, handgrip strength/body mass index, and CT parameter (psoas muscle attenuation or psoas muscle volume index). Multivariate model 3 includes age, type of operation, geriatric nutritional risk index, estimated glomerular filtration rate, brain natriuretic peptide, delirium and duration requiring mechanical ventilator, 6‐min walking distance, and CT parameter (psoas muscle attenuation or psoas muscle volume index).

CI, confidence interval; OR, odds ratio; SD, standard deviation.

## Discussion

The main findings of this study were the identification of preoperative CT‐derived low PMA as an indicator of delayed attainment of autonomous walking following cardiovascular surgery. Our research findings substantiated a robust association between PMA and the delayed achievement of independent walking after cardiovascular surgery, even after adjusting for the effects of age, type of operation, renal function, nutritional status, severity of heart failure, delirium incidence, and duration requiring mechanical ventilation, as well as preoperative physical fitness such as SPPB, GS/BMI, and 6MWD. To our knowledge, this is the first study to demonstrate that preoperative CT‐derived skeletal muscle assessment in patients undergoing cardiovascular surgery can predict the delayed attainment of independent walking after surgery. We are likely to consider PMA as a clinical and validated indicator for predicting the delay in independent walking post‐cardiovascular surgery because it is calculated from routinely taken CT images for pathophysiologic evaluation and stands as an objective measure unswayed by patient symptoms or eagerness for assessments.

Previous studies reported that the prevalence of sarcopenia in older patients undergoing cardiovascular surgery ranges from 19% to 27%.^12^ Within the realm of cardiovascular surgery, which is renowned for its invasiveness, a primary concern is delayed rehabilitation progress and suboptimal functional recovery in older patients with sarcopenia. Delays in achieving postoperative independent walking, a pivotal indicator of recovery, are inevitably associated with extended hospital stay and an elevated risk of mortality and readmission.^5^, ^6^, ^7^, ^8^ The primary endpoint of the present study was the ability of patients to walk independently within 4 days after surgery. This criterion is based on evidence from multicentre surveys conducted in Japan^26^ and the United States,^28^ making it a widely applicable benchmark for research. Notably, an analysis of the medical records of 496 797 patients who underwent coronary artery bypass surgery in the United States revealed that 53% of the patients were discharged within 5 days post‐surgery,^28^ making the goal of independent walking within 4 days post‐surgery reasonable. Although it is imperative for surgeons to integrate sarcopenia management into clinical practice to enhance risk prediction, patient selection, and postoperative care,^29^ the practicality of this endeavour for pre‐cardiovascular surgery patient populations remains limited.^30^ The speculated reasons include the time‐intensive nature of such assessments, constraints on available resources, and inherent risks due to maximal effort evaluations of physical fitness. However, preoperative CT imaging has already become an integral component of routine clinical practices for patients undergoing cardiovascular surgery. Its role is to assess surgical risks and enhance surgical planning.^9^ In recent years, CT has emerged as the gold standard for assessing skeletal muscle, with their strong correlation and interchangeability to magnetic resonance imaging (MRI).^1^, ^31^ MRI is widely recognized as one of the most advanced and dependable imaging techniques for scrutinizing skeletal muscle, capable of concurrently revealing qualitative abnormalities like muscle breakdown, oedema, and intramuscular fatty tissue/fibrosis.^32^ However, the high cost and complexity is problematic, and it is not included in a routine examination in cardiovascular surgery. Employing existing thoracoabdominal CT scans for skeletal muscle analysis not only enables the prediction of delayed attainment of independent walking after surgery, but also mitigates the need for additional radiation exposure and reduces medical costs for cardiovascular surgery patients. Moreover, in elective surgery cases, patients often have a period of approximately 1 to 2 months between the completion of preoperative CT evaluations and the actual surgery. Consequently, the ability to anticipate delays in the acquisition of independent walking following cardiovascular surgery opens the door to therapeutic intervention, such as prehabilitation, aimed at enhancing postoperative outcomes.^30^, ^33^

An analysis comparing the predictive capabilities of CT‐derived parameters and preoperative physical fitness in patients undergoing cardiovascular surgery revealed that PMA, signifying muscle quality, demonstrated the highest predictive efficacy for the delayed attainment of independent walking following cardiovascular surgery. Previous studies have documented that subpar psoas muscle quality, as determined by CT imaging, is associated with deficient muscle function at discharge and a more unfavourable prognosis, a finding that aligns with our study.^25^, ^34^ However, these investigations did not consider the influence of confounding factors such as preoperative‐assessed physical function, perioperative data, and nutritional status. We posit that the novelty of our study lies in the strong association between PMA and delayed achievement of independent walking following cardiovascular surgery. After considering the influence of the unadjusted factors in previous studies, a noteworthy aspect of our research is the introduction of postoperative early walking delay as a pioneering outcome measure. With respect to PMA being strongly associated with perioperative cardiovascular surgery, MA is a better indicator of muscle function and predictor of future mobility limitations than muscle mass.^35^, ^36^ MA is an indicator of intramuscular fat deposition^37^ and is associated with insulin resistance,^38^ inflammatory cytokine production,^39^ and postoperative complications.^40^, ^41^ Thus, as PMA reflects not only physical and mechanical dysfunction of the skeletal muscle, but also metabolic dysfunction,^42^ we speculate that it may be a stronger indicator of vulnerability to surgical invasion. As for one of the focal points of this study (the potential improvement in muscle quality during the preoperative waiting period), previous research focusing on community‐dwelling and institutionalized frail older males and females has shown increased muscle density with twice‐weekly resistance training.^43^, ^44^

With regard to the clinical application of results of the present study, preoperative PMA assessment via CT imaging plays an important role for determining the eligibility for preoperative rehabilitation. The beneficial effects of prehabilitation, comprising physical exercise and nutritional enhancement before surgery, on postoperative outcomes such as reduced hospital and intensive care unit stay, increased walking distance and gait speed, and reduced pulmonary complications have already been documented in previous studies.^33^, ^45^ However, these interventions have not yet been incorporated into routine clinical practice for patients undergoing cardiovascular surgery. The study's findings will help propagate the notion that preoperative rehabilitation, aimed at improving postoperative outcomes, should be specifically applied to patients identified with low PMA through preoperative CT skeletal muscle assessment.

Conversely, PMVI was not associated with delayed achievement of independent walking after cardiovascular surgery. Earlier investigations exploring the link between muscle mass ascertained using CT imaging and clinical outcomes have yielded inconclusive findings in patients undergoing cardiovascular procedures. Muscle mass correlates poorly with muscle strength and, more generally, muscle function. Therefore, the substantial decline in muscle function with ageing is more strongly dependent on muscle quality than on muscle mass.^35^, ^36^ These results support our findings that muscle mass is not associated with delayed postoperative walking acquisition.

This study has several limitations. First, the study was conducted at a single Japanese institution and the study design was retrospective, thereby limiting the generalizability of our findings to a broader international population. Therefore, further large‐scale studies are required to confirm these findings. Second, because of the small sample size, we were unable to analyse the data according to surgery type. As the pathophysiology and postoperative management strategies differ, it would be desirable to conduct the analysis separately for valvular heart disease, ischaemic heart disease, and aortic disease. Third, skeletal muscle analysis (i.e., segmentation) with CT uses only the average HU from manual analysis. However, fully automated analysis software has been developed in recent years, and it is desirable to use software that does not require manual labour in the future.

## Conclusions

Our study revealed a strong association between PMA, a marker of CT‐derived muscle quality, and the postoperative delay in achieving independent walking in patients who underwent cardiovascular surgery. Furthermore, PMA was shown to be a better predictor of delay in the postoperative acquisition of independent walking than physical function or muscle mass. The technique to obtain information on muscle quality during the time period before surgery may be an option for timely therapeutic intervention in patients who may have delayed acquisition of independent walking after surgery.

## Funding

This work was supported in part by research funding from Hyogo Medical University (to R.M.) and the Hyogo Medical University Graduate School of Rehabilitation Science (to K.S.).

## Conflicts of interest

The authors declare no conflict of interest.
